# Obstructive sleep apnea is associated with coronary microvascular dysfunction: A systematic review from a clinical perspective

**DOI:** 10.1111/jsr.13046

**Published:** 2020-04-15

**Authors:** Rui‐Heng Zhang, Wei Zhao, Lin‐Ping Shu, Nan Wang, Yao-Hua Cai, Jin‐Kui Yang, Jian‐Bo Zhou, Lu Qi

**Affiliations:** ^1^ Beijing Tongren Hospital Capital Medical University Beijing China; ^2^ Department of Geriatrics Beijing Tongren Hospital Capital Medical University Beijing China; ^3^ Department of Endocrinology Beijing Tongren Hospital Capital Medical University Beijing China; ^4^ Department of Epidemiology School of Public Health and Tropical Medicine Tulane University New Orleans LA USA

**Keywords:** coronary flow reserve, meta‐analysis, obstructive sleep apnea

## Abstract

There is now increasing evidence demonstrating that obstructive sleep apnea (OSA) contributes to microvascular disorder. However, whether OSA is associated with impaired coronary flow reserve is still unclear. Therefore, we conducted this systematic review and meta‐analysis to summarize current evidence. In a systematic review, PubMed, Embase, the Cochrane Library and Web of Science were searched; five observational studies fulfilled the selection criteria and were included in this study. Data were extracted from selected studies and meta‐analysis was performed using random‐effects modelling. In all, 829 OSA patients and 507 non‐OSA subjects were included and assessed for coronary flow reserve (CFR), the clinical indicator of coronary microvascular dysfunction (CMD). For all studies, OSA was significantly associated with reduced CFR. The pooled weighted mean difference (WMD) of CFR was −0.78 (95% confidence interval [CI] −1.25 to −0.32, *p* ＜ 0.001, I^2^ = 84.4%). The difference in the apnea–hypopnea index (AHI) between studies can explain 89% of heterogeneity (coef = −0.05, 95% CI −0.12 to 0.02, *p* = .078) in a meta‐regression, indicating the CFR tended to negatively correlate with severity of OSA. The Egger regression test did not show statistical significance (*p* = .49). In conclusion, there are plausible biological mechanisms linking OSA and CMD, and the preponderance of evidence from this systematic review suggests that OSA, especially severe OSA, is associated with reduced CFR. Future studies are warranted to further delineate the exact role of OSA in CMD occurrence and development in a prospective setting.

## 1 INTRODUCTION

Coronary microvascular dysfunction (CMD) is proposed to be a contributor to the signs and symptoms of myocardial ischaemia, which results from a limited microvascular vasodilator capacity under vasodilator stimuli (Crea, Camici, & Bairey Merz, 2014). Coronary flow reserve (CFR), quantified as the ratio of hyperaemic to rest myocardial blood flow, is a functional measure of large‐ and small‐vessel ischaemia, and in the absence of overt coronary artery disease (CAD), is a marker of CMD (Taqueti et al., 2018). A CFR ≤ 2.0 provides the optimal prognostic cut‐off value for CMD (Cortigiani et al., 2019). On the basis of the clinical settings in which it occurs, Camici classified coronary microvascular dysfunction into four types: (a) CMD in the absence of myocardial diseases and obstructive CAD ( cardiac X syndrome for example), (b) CMD in myocardial diseases, (c) CMD in obstructive CAD and (d) iatrogenic CMD (Camici & Crea, 2007). It has been estimated that up to 40% of typical angina‐like chest pain exhibits no angiographic obstructive CAD (Patel et al., 2010), with two‐thirds of all patients having some sort of CMD. Moreover, CMD is associated with adverse cardiovascular outcomes (Taqueti et al., 2018). In a large prospective study, CFR was a powerful incremental predictor of major adverse cardiac events (hazard ratio [HR] 0.80 [95% CI 0.75–086] per 10% increase in CFR) (Murthy et al., 2014). Also, CMD can explain the persistence of anginal symptoms in 20% of patients 12 months after revascularization, despite successful myocardial revascularization (Cohen et al., 2011).

Obstructive sleep apnea (OSA), characterized by recurrent episodes of hypoxaemia and reoxygenation, is a common sleep‐disordered breathing condition frequently found in the general population (Young et al., 1993). It is estimated that the overall population prevalence ranges from 9% to 38% at ≥5 events/hr, apnea–hypopnea index (AHI), and is higher in men (Senaratna et al., 2017). OSA is associated with higher risk of cardiovascular morbidity (Chan et al., 2019). However, there is still controversy over the underlying mechanism. In a meta‐analysis, positive airway pressure did not reduce risks of cardiovascular outcomes or death for patients with sleep apnea (obstructive and central) (Yu et al., 2017). Recent evidence even suggested an increased all‐cause and cardiovascular mortality in patients with central sleep apnea, in systolic heart failure, who received adaptive servo‐ventilation (Cowie et al., 2015). Thus, deciphering the mechanisms, causal relationships and potential therapeutic targets in the links between OSA and cardiovascular morbidity is urgently needed.

Obstructive sleep apnea has been independently associated with endothelial dysfunction, which may explain the increased risk of cardiovascular and all‐cause mortality in this population (Hoyos, Melehan, Liu, Grunstein, & Phillips, 2015). In this study, we performed a systematic review and meta‐analysis to evaluate the association between OSA and CMD. We focus on CMD in the absence of obstructive CAD and myocardial disease, defined as impaired CFR.

## 2 MATERIALS AND METHODS

### 2.1 Study design

We systematically reviewed observational studies that described the association between OSA and CFR. Included and excluded studies were collected following the Preferred Reporting Items for Systematic Reviews and Meta‐Analyses (PRISMA) flow diagram (Kahnert et al., 2017). This meta‐analysis was performed according to the Meta‐analysis Of Observational Studies in Epidemiology (MOOSE) guidelines (Kuziemski, Pienkowska, Slominski, Jassem, & Studniarek, 2015).

### 2.2 Search strategy

A systematic search was performed by two authors (Zhang, Zhao) independently on April 26, 2019. We searched MEDLINE (published between 1946 and April 26, 2019), Embase (published 1974 to April 26, 2019), Web of Science (published between 1986 and 2019) and the Cochrane Library (no time restriction) using the free text terms (‘Coronary Microvascular Dysfunction’ OR ‘Coronary Circulation’ OR ‘Coronary microcirculation dysfunction’ OR ‘Coronary Vessels’ OR ‘Myocardial Perfusion’ OR ‘Coronary flow reserve’ OR ‘Coronary microcirculation’ OR ‘Coronary flow’ OR ‘velocity reserve’ OR ‘Coronary vasoreactivity’ OR ‘Vasodilator reserve’) AND (‘Sleep Apnea Syndromes’ OR ‘obstructive sleep apnea hypopnea syndrome’ OR ‘OSA’ OR ‘SHS’ OR ‘OSAHS’). Relevant articles from the reference lists of the retrieved articles were also searched.

Search results were restricted to only articles involving humans and in English. No other restrictions applied in this procedure. Search results were input in EndNote X7 and duplicate literatures were removed automatically.

### 2.3 Inclusion and exclusion criteria

Two authors (R.‐H.Z. and W.Z.) independently screened all studies by title and abstract, and considered them for inclusion if they met the following criteria: (a) English full‐text available for assessment and (b) sufficient data on CMD between an OSA group and non‐OSA group. We excluded studies that met any of the following criteria: (a) had insufficient data for methodological quality assessment, (b) had participants with obstructive coronary artery diseases or myocardial disease and (c) were reviews, editorials, letters, abstracts, case reports or practice guidelines.

The Newcastle‐Ottawa Scale (NOS) was used for assessing the quality of non‐randomized studies in a meta‐analysis (Wells et al., 2000). In general, we used this scale (with a maximum of nine stars) to evaluate each study on three aspects: selection of participants, comparability of study groups and the ascertainment of outcomes of interest.

### 2.4 Data extraction

For all studies, we extracted information on study size, participants’ inclusion and exclusion criteria, AHI and clinically important covariables (age, diabetes, body mass index (BMI) and hypertension), method of diagnosis of CFR and OSA, and main outcomes.

### 2.5 Data synthesis and analysis

The outcome measure of this meta‐analysis was the CFR among individuals with OSA compared with those without OSA. Heterogeneity was assessed by the *I* ^2^ statistic values: ∼25% represents low heterogeneity, ∼50% represents medium heterogeneity and ∼75% represents high heterogeneity. The fixed‐effect model was applied when it was slight (*I* ^2^ ≤ 50%); otherwise, the random‐effect model was used. Publication bias was assessed through a funnel plot and Egger's test. All statistical tests were two‐sided and used a significance level of *p* < .05. All statistical analyses were performed in STATA 12.0 (StataCorp).

## 3 RESULTS

### 3.1 Characteristics of included studies

**FIGURE 1:**
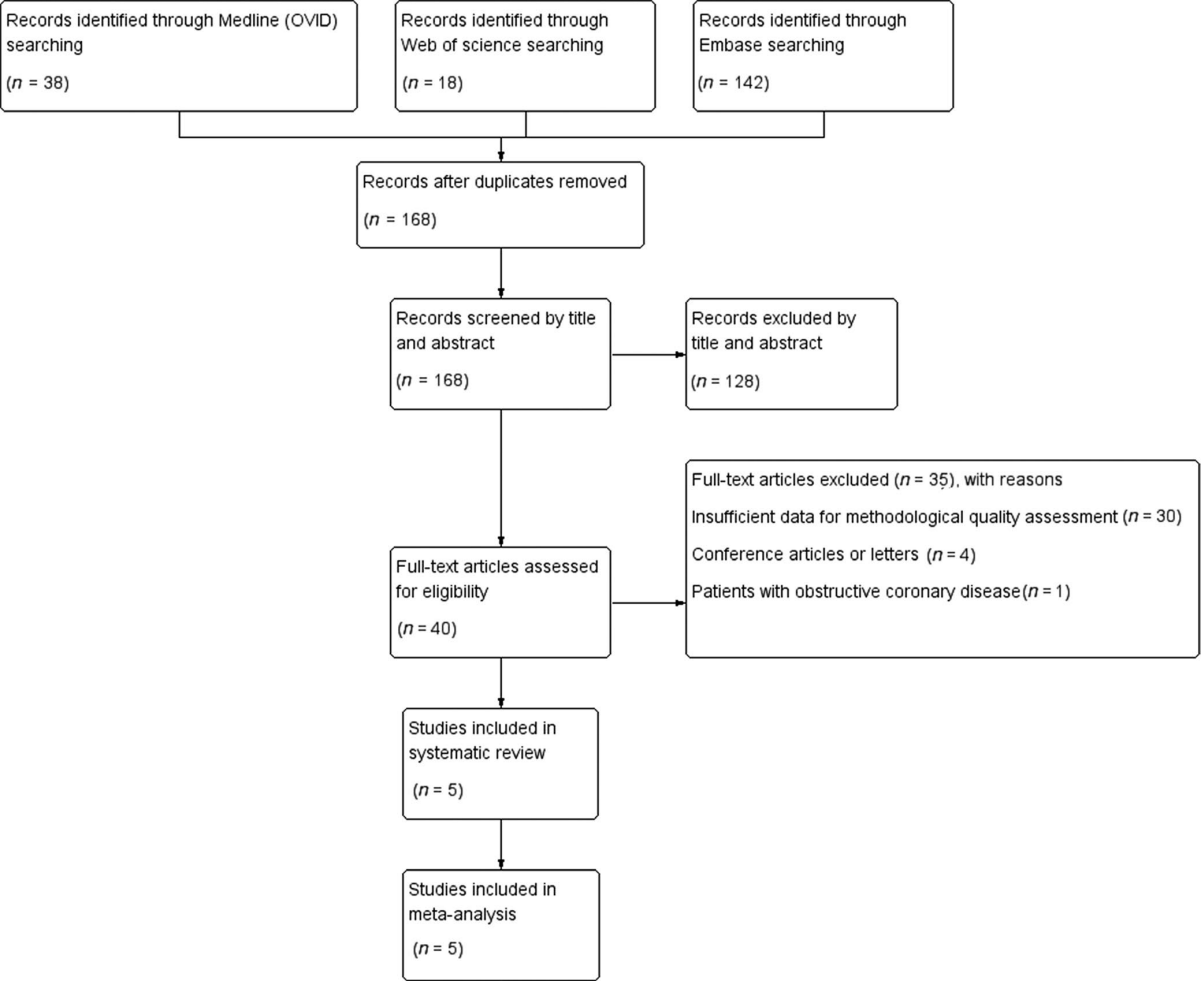
Flow chart summarizing study identification and selection

A flow diagram of study selection is presented in Figure 1. In brief, the MedLine (OVID), EMBASE and Web of Science search yielded 38, 142 and 18 hits, respectively. After duplicates were removed, 168 articles were excluded by title and abstract. A total of five studies (Bozbas, Eroglu, Ozyurek, & Eyuboglu, 2017; Butt et al., 2011; Cassar et al., 2014; Obase et al., 2011; Wang et al., 2014) with 507 non‐OSA participants and 829 OSA patients were included in this systematic review, and all included studies collected data from clinic‐based samples. Two studies (Cassar et al., 2014; Wang et al., 2014) utilized an invasive method, whereas others (Bozbas et al., 2017; Butt et al., 2011; Obase et al., 2011) utilized a non‐invasive method to evaluate coronary microcirculation. Specifically, only one study used an endothelium‐dependent vasodilator, acetylcholine (Cassar et al., 2014), to reach maximum coronary blood flow, whereas the others used endothelium‐independent vasodilators, dipyridamole (Bozbas et al., 2017; Butt et al., 2011) or adenosine triphosphate (Obase et al., 2011; Wang et al., 2014). In all, data on the coronary microcirculation of 1,336 participants were measured. Other patient characteristics of the included studies are presented in Table 1.

**TABLE 1:** Characteristics of included studies

Study	Cassar (2014)	Bozbas (2017)	Obase (2011)	Butt (2011)	Wang (2014)
Study design and location	Cross‐sectional (USA)	Cross‐sectional (Turkey)	Cross‐sectional (Japan)	Case–control (UK)	Cross‐sectional (China)
Study quality (assessed by NOS)	Selection: *** Comparability: * Outcome: *** Total: 7	Selection: *** Comparability: ** Outcome: *** Total: 8	Selection: ** Comparability: Outcome: ** Total: 4	Selection: **** Comparability: * Outcome: *** Total: 8	Selection: *** Comparability: * Outcome: *** Total: 7
Sample size (male %)	143 (32%)	61 (74%)	22 (82%)	72 (69%)	1,038
Participants	Inclusion: medical history of patients who had undergone polysomnography and invasive coronary vasomotor study. Exclusion: obstructive CAD, angiographic coronary artery >40% luminal diameter stenosis, heart failure with an ejection fraction <40%, valvular heart disease, stroke, or significant hepatic, renal or inflammatory disease within 6 months of the invasive study	Inclusion: patients who had symptoms of nocturnal snoring and/or excess daytime sleepiness and had undergone polysomnography. Exclusion: obstructive CAD, valvular heart disease, cardiomyopathy, heart failure, uncontrolled hypertension before study, chronic kidney disease, asthma, malignancy, central sleep apnea, and those using any vasoactive drugs	Inclusion: consecutive patients who had newly diagnosed OSA and healthy controls (asymptomatic, normotensive, non‐diabetic and non‐smokers) Exclusion: obstructive CAD, asthma	Inclusion: newly diagnosed OSA participants and community healthy controls. Exclusion: diabetes mellitus, hyperlipidaemia, obstructive CAD, known structural heart disease, left ventricular dysfunction, previous cerebrovascular event, malignancy, connective tissue or inflammatory disease, chronic infection, and hepatic or renal impairment	Inclusion: patients with chest pain, angiographically normal epicardial coronary arteries, and normal left ventricular function. Exclusion: obstructive CAD, angiographic coronary artery >50% luminal diameter stenosis, left ventricular hypertrophy, valvular heart disease or unstable angina
Age, years, mean ± *SD*	Non‐OSA: 47.2 ± 9.3 OSA: 50.4 ± 13.4	Non‐OSA: 51.3 ± 10.4 OSA: 50.6 ± 11.0	Non‐OSA: 54.0 ± 6.0 OSA group: 51 0.0 ± 8.0	Non‐OSA group: 47 ± 9 OSA group: 49 ± 10	Non‐OSA group: 62.7 ± 11.5 Mild‐to‐moderate OSA: 62.7 ± 11.4 Severe OSA: 63.6 ± 11.7
Diagnosis method of OSA	Polysomnography AHI ≥ 5	Polysomnography AHI ≥ 5	Polysomnography AHI ≥ 5	Polysomnography with a diagnosis of moderate‐severe OSA AHI > 15	Polysomnography AHI ≥ 5
Apnea–hypopnea index (*n*/hr)	OSA group: 13 (8,27) [median (Q1,Q3)]	OSA group: 21.7 (11.7–42.2) [median (Q1,Q3)]	OSA group: 40.6 ± 14.5 [mean ± *SD*]	OSA group: 36 ± 20 [mean ± *SD*]	NA
Hyperlipidaemia, *N* (%)	Non‐OSA: 24 (59%) OSA: 73 (73%)	NA	**Non‐OSA: 6 (55%)** **OSA: (0%)**	Non‐OSA: 0 (0%) OSA: 0 (0%)	Non‐OSA: 195 (48.4%) Mild‐to‐moderate OSA: 196 (48.4%) Severe OSA: 124(49.8%)
Current smoker, *N *(%)	Non‐OSA: 5 (12%) OSA: 15 (15%)	Non‐OSA: 11 (28.8%) OSA: 38 (84.4%)	NA	NA	Non‐OSA: 60 (14.9%) Mild‐to‐moderate OSA: 60 (15.5%) Severe OSA: 39 (15.7%)
Hypertension, *N* (%)	**Non‐OSA: 12 (29%)** **OSA: 60 (59%)**	Non‐OSA: 6 (37.5%) OSA: 14 (31.1%)	**Non‐OSA: 0 (0%)** **OSA: 7 (63.6%)**	Non‐OSA: 0 (0%) OSA group: 0 (0%)	Non‐OSA group: 110 (27.3%) Mild‐to‐moderate OAS: 122 (31.6%) Severe OSA: 83 (33.3%)
Diabetes, *N* (%)	Non‐OSA: 4 (10%) OSA: 14 (14%)	Non‐OSA group: 0 (0%) OSA group: 3 (6.7%)	Non‐OSA group: 0 (0%) OSA group: 4 (36.4%)	Non‐OSA group: 0 (0%) OSA group: 0 (0%)	Non‐OSA group: 59 (14.6%) Mild‐to‐moderate OSA: 58 (15.0%) Severe OSA: 38 (15.3%)
Diagnosis method of CFR	Invasive method: Doppler guide‐wire within a coronary infusion catheter was positioned in the mid portion of the left anterior descending coronary artery. Endothelium‐dependent stressor: acetylcholine	Non‐invasive method: transthoracic Doppler echocardiographic examinations were performed in mid to distal left anterior descending artery. Endothelium‐independent stressor: dipyridamole	Non‐invasive method: transthoracic Doppler echocardiographic examinations were performed in left anterior descending artery. Endothelium‐independent stressor: adenosine triphosphate	Non‐invasive method: quantitative myocardial contrast echocardiography. Endothelium‐independent stressor: dipyridamole	Invasive method: Doppler guide‐wire within a coronary infusion catheter was positioned in the left anterior descending coronary artery. Endothelium‐independent stressor: adenosine triphosphate
Main outcomes	Non‐OSA group CFR: 3.1 ± 0.7 OSA group CFR: 3.1 ± 0.9	Non‐OSA group CFR: 2.74 ± 0.62 OSA group CFR: 2.24 ± 0.46	Non‐OSA group CFR: 3.2 ± 0.7 OSA group CFR: 2.2 ± 0.9	Non‐OSA group CFR: 3.5 ± 1 OSA group CFR: 2 ± 1	Non‐OSA group CFR: 3.2 ± 0.7 OSA group CFR: 2.8 ± 0.9

Note

Bold: statistically significant between groups.

Abbreviations: CAD, coronary artery disease; CFR, coronary flow reserve; CMD, coronary microvascular dysfunction; NOS, The Newcastle‐Ottawa Scale;OSA, obstructive sleep apnea; *SD*, standard difference.

The star mark ‘*’ represents one score, which is universally used in NOS assessing.

### 3.2 OSA and CFR

**FIGURE 2:**
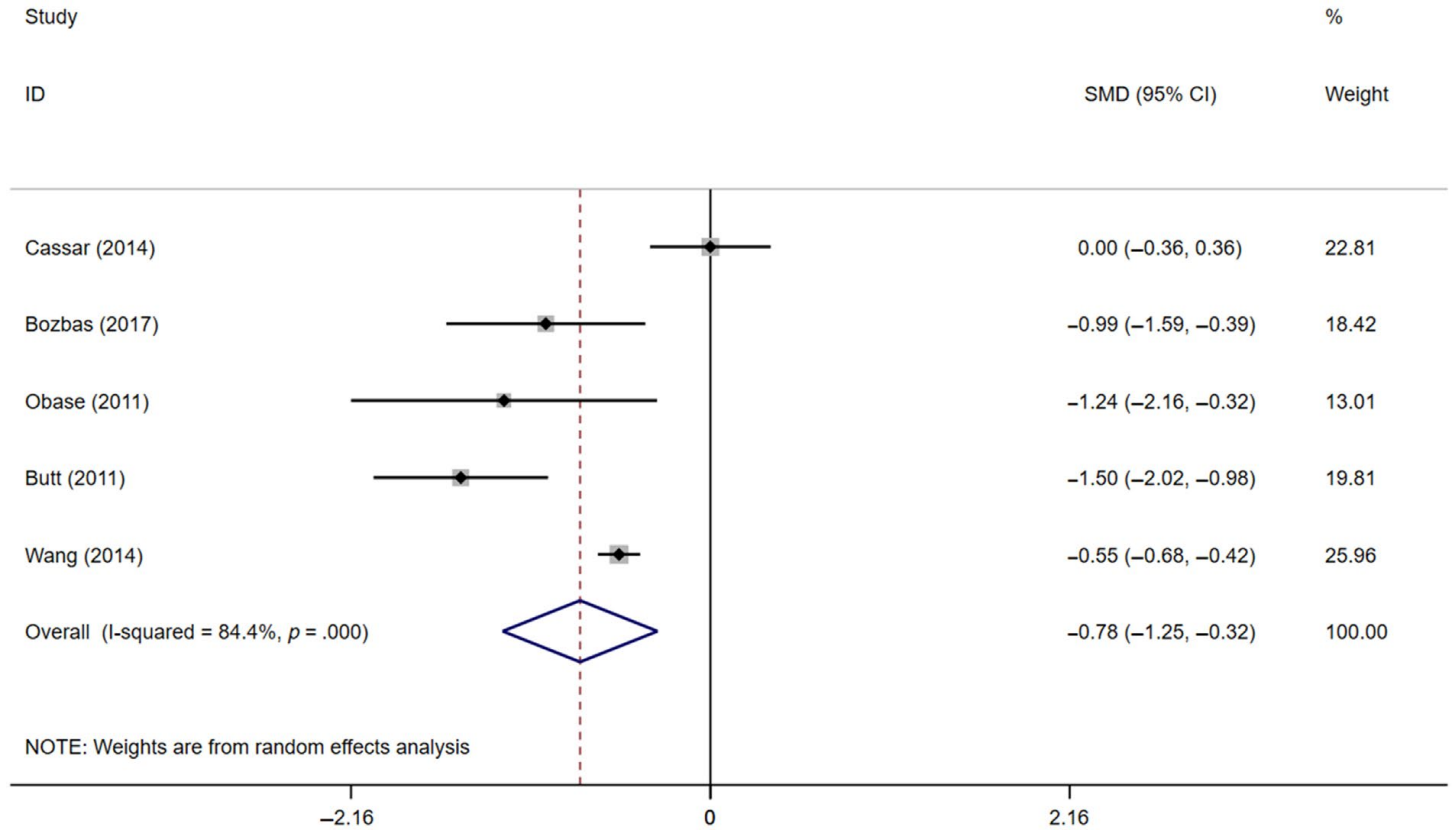
Forest plot and pooled estimates of the effect of obstructive sleep apnea (OSA) on coronary flow reserve (CFR). WMD, weighted mean difference

**FIGURE 3:**
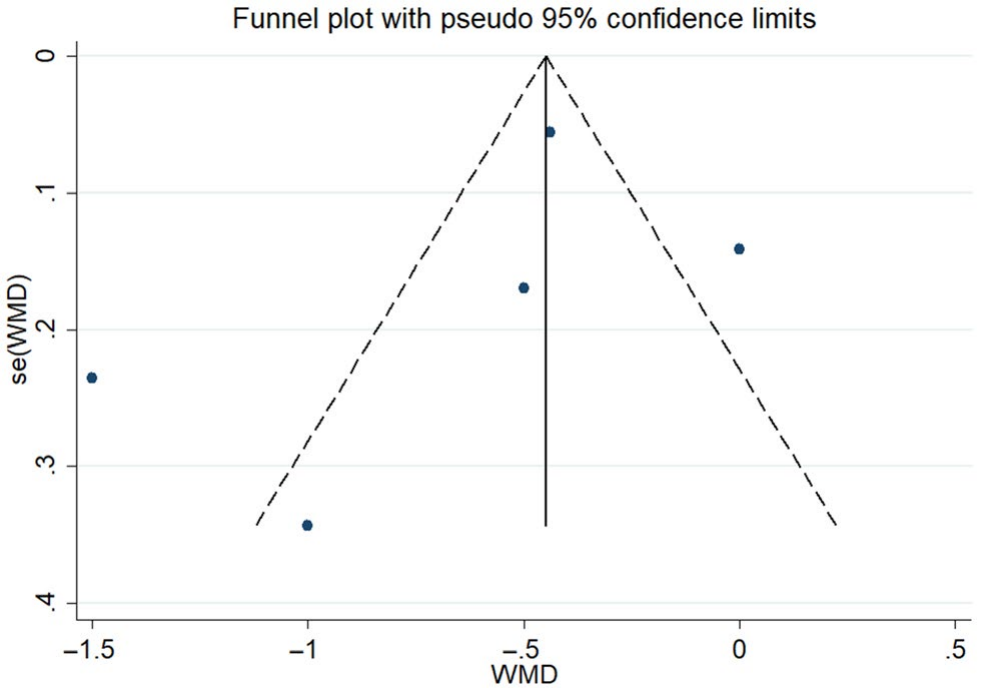
Funnel plot of standard error by weighted mean difference (WMD) for coronary flow reserve (CFR). Egger's regression test: *p*‐value = .49. se, standard error

Obstructive sleep apnea was significantly associated with lower CFR. Compared to participants without OSA, the pooled weighted mean difference (WMD) of CFR was −0.78 (95% CI −1.25 to −0.32, *p* ＜ .001, *I*_2_ = 84.4%; Figure 2) in OSA patients. Remarkably, we detected high heterogeneity between studies, which cannot be explained by either the methods of diagnosis of CFR, or stressors for reaching hyperaemic blood flow. To explore the potential source of heterogeneity, we performed the meta‐regression. Differences in the AHI between studies can explain 89% of heterogeneity (coef = −0.05, 95% CI −0.12 to 0.02, *p* = .078), indicating that the CFR tended to negatively correlate with severity of OSA. The funnel plot suggests potential publication bias (Figure 3) in two studies that are outliers of the other three studies, and the Egger regression test did not find statistical significance (*p* = .49). Given the limited number of studies, we can't rule out the possibility of publication bias.

Previous studies revealed that AHI, time with SpO_2_ < 90% and other severity parameters have different extents of deleterious effects on cardiovascular diseases (Aurora, Crainiceanu, Gottlieb, Kim, & Punjabi, 2018; Gami et al., 2013). Among all participants (including healthy and OSA patients), one study exhibited that AHI and time with SpO_2_ < 90% were negatively associated with CFR (*β* = −0.3, *p* = .008; *β* = −0.4, *p* = .01) (Bozbas et al., 2017). However, evidence also indicated that CFR was not worse in OSA patients whose SpO_2_ < 90% time exceed 10% of sleep time (Cassar et al., 2014). Moreover, an included study showed hypertension alone did not alter CFR (*p* = .87) (Butt et al., 2011). When including age, BMI, OSA, diabetes, hypertension, smoking and chronic obstructive pulmonary disease in a multivariate model, only OSA was significantly associated with CFR (*β* = −0.38, *p* = .009) (Bozbas et al., 2017).

## 4 DISCUSSION

The coronary arterial system is composed of three compartments (epicardial coronary arteries, prearterioles and arterioles), each governed by distinct regulatory mechanisms (Camici & Crea, 2007) In the absence of obstructive stenoses, the epicardial arteries (>500 µm in diameter) and the prearterioles (200 µm < vessels <500 µm in diameter) serve mainly as conductance vessels that maintain adequate perfusion pressure in the distal arteriolar bed (Herrmann, Kaski, & Lerman, 2012). The arterioles (<200 µm) are the predominant regulatory component of the coronary circulation. Endothelium‐dependent vasoreactivity prevails in the larger arterioles (100–200 µm in diameter) and translates flow‐related stimuli into vasomotor responses. They also react to neural factors, such as acetylcholine. Medium‐sized arterioles (40–100 µm in diameter) react predominantly to intraluminal pressure changes, sensed by stretch receptors located in vascular smooth muscle cells. Finally, the tone of the smaller arterioles (vessels < 40 µm in diameter) is modulated by the metabolic activity of the myocardium, such as reaction to adenosine (Herrmann et al., 2012).

Contrary to the epicardial coronary vasculature, the coronary microcirculation has remained elusive to conventional imaging techniques. The retinal vascular bed is the only microvasculature that can be directly visualized non‐invasively with retinal photography (Ding et al., 2010), and it is structurally and functionally similar to microvasculature elsewhere in the body (Liew, Wang, Mitchell, & Wong, 2008). Recent evidence demonstrated a significant association of OSA with retinal microvascular signs after comprehensive adjustments (Lin et al., 2016; Shankar et al., 2013). In Tahrani's cohort study of adults with type 2 diabetes, estimated glomerular filtration rate declined faster in patients with OSA, demonstrating a persistent deleterious effect on the renal microvasculature (Tahrani et al., 2013). In this systematic review and meta‐analysis, with a total of five unique observational studies with aggregate data on 1,336 individuals and 829 cases of OSA, the preponderance of evidence suggests an association between OSA and CMD. OSA conferred a WMD decrease of 0.78 for CFR, and CFR tended to negatively correlate with severity of OSA. Our study adds more evidence regarding the damage by OSA to the microcirculation in the heart.

There are several mechanisms that might explain the association between OSA and CMD. The repetitive episodes of reoxygenation after hypoxaemia in OSA patients resemble ischaemia/reperfusion injury, which is associated with excessive production of reactive oxygen species (ROS) (Badran, Ayas, & Laher, 2014). ROS can directly change a protein conformation, exposing the catalytic site of matrix metalloproteinases (Franczak et al., 2019) and thus causing inward eutrophic remodelling in resistance arteries (Chelladurai, Seeger, & Pullamsetti, 2012; Martinez‐Lemus, Zhao, Galiñanes, & Boone, 2011). In addition, increased peroxynitrite production was found in the microvascular walls of patients with OSA, indicating overproduction of nitrous oxide (NO) and superoxide in the endothelial environment (Patt et al., 2010). These results explained the observations that OSA was associated with suppressed circulating NO and flow‐mediated dilatation (Ip et al., 2000; Wang et al., 2017).

Some limitations of our study must be taken into consideration. Firstly, high heterogeneity was detected in this meta‐analysis. It is plausible that this high heterogeneity reflects differences in the participants, various testing techniques for CFR and OSA, severity of OSA and the difference in morbidities among participants (hypertension, obesity, diabetes, etc.). Among the five studies, four studies recruited participants who were suspected of having OSA and had this confirmed by polysomnography, whereas one study recruited patients with chest pain and angiographically normal epicardial coronary arteries (see Table 1). Moreover, the definition of OSA varied across the studies. All studies defined OSA by an AHI ≥ 5/hr. However, some of the studies used 3% desaturation to score hypopnea (Bozbas et al., 2017; Obase et al., 2011), others 4% (Wang et al., 2014). Two of the studies did not note the cut‐off for oxygen desaturation at all (Butt et al., 2011; Cassar et al., 2014). Furthermore, only two studies used the reference standard for measuring CFR (see Table 1), whereas three used transthoracic Doppler echocardiography (TTDE). Although TTDE is a widely used non‐invasive technique for measuring CFR and demonstrates good agreement with invasive intracoronary Doppler flow wire (Caiati et al., 1999; Hozumi et al., 1998; Lethen, P Tries, Kersting, & Lambertz, 2003), this non‐invasive technique is position and image dependent (Herrmann et al., 2012). Remarkably, we found that CFR tended to negatively correlate with severity of OSA, indicating that the severity of OSA contributed to the majority of between‐studies heterogeneity.

Secondly, deciphering the mechanisms, causal relationships and potential therapeutic targets in the links between OSA and CMD has not been an easy task, in part because of the constellation of comorbidities that characterize a large proportion of OSA patients. In this review, information on some important confounders (e.g., smoking, hyperlipidaemia, the severity of OSA, antihypertensive agents, anti‐ischaemia agents and lifestyle changes) was often missing. Hypertension and diabetes commonly coexisted in OSA patients in some studies. They are well‐known deleterious factors for coronary microcirculation (Erdogan et al., 2007; Prior et al., 2005). The small sample sizes may be the reason that the included studies did not provide evidence that hypertension or diabetes altered the association of OSA and CFR.

Thirdly, the original studies were all of a cross‐sectional design, and there is no prospective study concerning the longitudinal relationship of OSA and CFR decline. Furthermore, only a few small, non‐randomized controlled trials exhibited a protective effect of continuous positive airway pressure (CPAP) therapy on coronary microcirculation (Butt et al., 2011; Obase et al., 2011), which were far from conclusive. Thus, a direct relationship between OSA and impaired CFR cannot be demonstrated.

## 5 CONCLUSION

In conclusion, there are plausible biological mechanisms linking OSA and CMD, and the preponderance of evidence from this systematic review suggests that OSA, especially severe OSA, is associated with lower CFR. However, the results of studies published on this subject are inconclusive and, thus, further research is needed to delineate the exact role of OSA in the occurrence and development of CMD in a prospective study. In addition, a well‐designed RCT is urgently needed to examine the protective effect of CPAP therapy on coronary microcirculation.

## CONFLICT OF INTEREST

All authors declare that they have no conflict of interest.

## AUTHOR CONTRIBUTIONS

RHZ and WZ contributed equally to the study design, literature search, statistical analysis, interpretation of data, and drafting the report; LPS, NW and YHC contributed to statistical analysis, interpretation of data; JBZ and LQ contributed to study design and review. JBZ is the guarantor of the paper, taking responsibility for the integrity of the work.

## ETHICAL APPROVAL

This article does not contain any studies with human participants or animals performed by any of the authors.
